# 

**Published:** 1872-05

**Authors:** 


### INDEX OF SUBJECTS.

### ORIGINAL COMMUNICATIONS.

Variola........257
Functional Diseases of the Eye........260
Gleanings from the Minutes of the Medical Society of the City of Wheeling, and County of Ohio, West Virginia........265
Robson on the Morbid Adhesion of the Placenta, Etc........268
Case of Foreign Body External to the Knee-Joint........270
Complete False Anchylosis of Hip Joint—Cure........271
Bromide of Ammonia in Dysmenorrhœa—Correspondence........275
Radical Cure of Hæmorrhoids........278
Mississippi State Medical Association........282

### SELECTIONS.

Diseases of the Rectum Stimulating Uterine Disease, 286; On Spurious Consumption, 288; Cervical Abscesses in Children, 290; Recent Treatment of Aural Diseases, 292; Notes on Mucous Disease, 295; Treatment of Hysterical Mania, 297; Treatment of Enlarged Spleen (Ague Cake), 298.

### EXTRACTS AND GLEANINGS.

Spinal Cupping in Tedious Labor, 299; From our Private Prescription Book, 300; Bromide of Potassium in Epilepsy, 301; Clinical Treatment of Pneumonia, 302; Nitrate of Amyl in Colic and Enteralgia—Carbolic Acid Inhalations in Phthisis, 303; Diagnosis of Glaucoma—Art of Prescribing for Children, 304; On the Treatment of Bedsores, 305; Treatment of Chronic Diarrhœa, 306; Bromide of Potassium in Sick Headache—Belladonna as an Antigalactic—Diptheria and Scarlet Fever Treated by Ice, 307; Treatment of Gonorrhœa—Oil of Cade in Eczema, 309; Atomized Turpentine Water in Chronic Bronchitis and Consumption—Carbuncle Treated by Cups—Chloral in Cholera—Braun's Colpeurynter as a Tampon—Inunction in the Treatment of Disease, 310; Diptheritic Enteritis, 311; Glycerin in Putrid Sore Throat—Carlo Paresi's Method of Concealing the Taste of Cod-Liver Oil—Death from Syrup of Poppies, 312; Veratrum Viride in Inflammatory Affections—Phosphorized Ether in Depression, 313; Connection of Various Diseases, Variola—Chloral in the Opium Habit, 314; Post-Partem Dietectic Treatment—Chronic Otitis, 315; Nitrate of Amyl in Asthma, 316.

### EDITORIALS, REVIEWS, ETC.

Editorial........317
Books and Pamphlets........319

